# Errors in ‘BED’-Derived Estimates of HIV Incidence Will Vary by Place, Time and Age

**DOI:** 10.1371/journal.pone.0005720

**Published:** 2009-05-28

**Authors:** Timothy B. Hallett, Peter Ghys, Till Bärnighausen, Ping Yan, Geoff P. Garnett

**Affiliations:** 1 Imperial College London, London, United Kingdom; 2 Joint United Nations Programme on AIDS (UNAIDS), Geneva, Switzerland; 3 Africa Centre for Health and Population Studies, University of KwaZulu-Natal, Durban, South Africa; 4 Public Health Agency of Canada, Ottawa, Canada; Yale University, United States of America

## Abstract

**Background:**

The BED Capture Enzyme Immunoassay, believed to distinguish recent HIV infections, is being used to estimate HIV incidence, although an important property of the test – how specificity changes with time since infection – has not been not measured.

**Methods:**

We construct hypothetical scenarios for the performance of BED test, consistent with current knowledge, and explore how this could influence errors in BED estimates of incidence using a mathematical model of six African countries. The model is also used to determine the conditions and the sample sizes required for the BED test to reliably detect trends in HIV incidence.

**Results:**

If the chance of misclassification by BED increases with time since infection, the overall proportion of individuals misclassified could vary widely between countries, over time, and across age-groups, in a manner determined by the historic course of the epidemic and the age-pattern of incidence. Under some circumstances, changes in BED estimates over time can approximately track actual changes in incidence, but large sample sizes (50,000+) will be required for recorded changes to be statistically significant.

**Conclusions:**

The relationship between BED test specificity and time since infection has not been fully measured, but, if it decreases, errors in estimates of incidence could vary by place, time and age-group. This means that post-assay adjustment procedures using parameters from different populations or at different times may not be valid. Further research is urgently needed into the properties of the BED test, and the rate of misclassification in a wide range of populations.

## Introduction

To date, HIV prevalence has been the main measure used in monitoring HIV epidemics, but it is neither timely nor easily interpreted, especially since antiretroviral treatment can increase prevalence without concomitant increases in the spread of the virus [1], [2], [3], [4]. A measure of incidence would provide a better tool to plan and evaluate HIV programmes [5], but cohort studies are prohibitively expensive and often unrepresentative. Mathematical models provide an indirect way to estimate incidence [6], [7], but a practical and valid method of measuring incidence from cross-sectional surveys would be ideal, and a number of assays have been developed in the hope of serving this purpose [8].

The underlying principle of these assays is that the immunological response to HIV evolves over the first months of infection, and by measuring the quantity, proportion or avidity of HIV antibody, recent infections can be discriminated from older ones. The most widely used of these assays is the BED capture enzyme immunosorbent assay (‘BED test’), in which the optical density varies according to proportion of IgG that is anti-HIV antibody [9]. A recent infection is usually defined as one for which the optical density of the test is less than 0.8, which corresponds to approximately 150–187 days after seroconversion [10], [11], [12] – in this article, this period is denoted 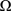. Assuming that all (or a known proportion of) the detected recent infections have occurred within a period 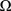 preceding the survey, the number of incidence infection occurring in the last year can be estimated [10].

BED-derived estimates of incidence have been compared with gold-standard measures of incidence in a range of settings [10], [13]. BED estimates are typically substantially too high [13], [14], leading to calls for caution in the use and interpretation of the test [15]. It has become clear that this is because the test misclassifies some individuals infected for more than one year as being recently infected [14]. Current guidelines [16] support using a post-assay correction calculation, using an empirical measurement of the fraction of individuals infected for more than a year are misclassified as recent (labelled 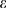 by Hargrove *et al.* [14]). It follows that the success of this correction procedure will be dependent on the accuracy of the value used for 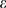 [17].


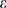
 has been measured in a small number of populations [13], [14], [17], [18], [19] and values vary between 1.7% (South Africa [17]) and 27% (Rwanda and Zambia [13]). Estimating incidence using the correction formulae and values of 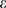 measured in one population to estimate incidence in another, had lead to seemingly unrealistically high estimates of incidence estimates in Cote d'Ivoire [18], South Africa [17], [20], Uganda [21] and Kenya [22], and unrealistically low estimates in Kwazulu-Natal in South-Africa [17]. There have been calls for further studies measuring 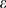 [15], [16], but if it is found that it varies widely, necessitating measurement in every population in which the BED test is used, then the usefulness of the BED test would be limited.

One key property of the BED test is the relationship between the chance that an infection is misclassified as recent (Proportion False Positive), and the time since infection (denoted *PFP(t)*). (Note that the 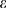
of Hargrove *et al.* is not time-variant.) This property has not been fully quantified, but there is mounting evidence that the chance of misclassification is higher for those with advanced infections. One reason is that the proportion of IgG that is HIV antibody could fall below the threshold in response to either the onset of opportunistic infections or treatment with antiretroviral therapy [23], [24], [25]. In small prospective studies, some individuals have been observed to revert to false positive result after months of infection. Treatment initiation also leads to regression past/towards the optical density cut-off [13], [24], [25]. Furthermore, a new study shows that the chance of misclassification is much higher for individuals indicated to start therapy and with low CD4 cell counts, than those with established infection but without symptoms [26]. In one study of post-partum women, the rate of misclassification was not associated with age or CD4 cell count (although there were low numbers of women with the lowest CD4 counts) [14], but this is not necessarily inconsistent with an up-turn in PFP many years after infection (fertility declines steeply with time infected with HIV [27], and most women in this study would probably have been infected in the previous few years). Another reason why misclassification could increase with time since infection would be if the individuals with fully developed immune-responses that are misclassified as recent live for longer than others. This is supported by observations of elite viral suppressors, that live for longer than those with higher viral loads, being more often misclassified as recent than others [23], [28].

To explore the influence that the relationship between PFP and time since infection could have on 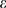 and ‘corrected’ BED estimates of incidence, we constructed a mathematical model describing HIV incidence, prevalence and the distribution of time since infection across a population, and represented a range of epidemics from six countries in sub-Saharan Africa (Kenya, Lesotho, Mozambique, Nigeria, Uganda and Zambia). We evaluated 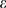 in the different settings, and compared the simulated incidence rates with the corresponding corrected BED-derived estimates that would arise from surveys of the modelled population, making alternative assumptions about how the PFP varies over longer times since infection. We then explored how reliably the BED test could track changes in incidence over time by modelling an instantaneous change in incidence by a factor 0.5, 0.75, 1.0, 1.25 or 1.5. Finally we calculated the required sample sizes in the surveys for a trend to be statistically significant.

## Methods

A system of partial differential equations was used to track numbers of susceptible and infected individuals in each sex and age-group over time (Text S1). Incidence rates in six African countries across all ages, 1985–2005, were calculated using Spectrum software [29] and UNAIDS estimates of prevalence [1], and were input to the model (Figure S1). The relative rate of incidence according to sex and age groups was based on recent empirical observations in eastern Zimbabwe [30] (Table S1) and it was assumed that this pattern is approximately constant over time. Net survival with HIV is assumed to be Weibull distributed and dependent on the age at infection [31] (Table S2). Background mortality rates (from causes other than HIV) and fertility rates are based on observation in African population in the pre-AIDS era [32] (Table S3).

**Figure 1:**
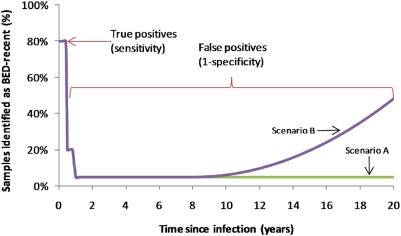
The influence of time since infection on the proportion of BED test results that would be positive. The pattern over the first year is informed by observational data, but pattern over the remaining time is uncertain and two hypothetical scenarios were used in our modelling (scenarios A and B).

To understand the properties of the BED test that determine the accuracy of derived incidence estimates, we define the ‘*BED response function*’ 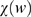 as the fraction of blood samples from individuals who are alive and have been infected for 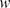 years that the BED test classifies/misclassifies as ‘recent’. As described above, the period after infection for which infections should be classified as recent by a perfect test is 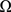. In this way, we write that the sensitivity of the test is: 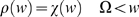, and the specificity of the test to infections that are 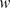 years old as: 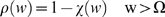. (For complete definitions of sensitivity and specificity using this notation, see Appendix.) The BED response function can be approximately parameterised from data for the first two years after infection (Figure 1). 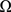 is taken to be 0.5 years; 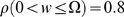, and 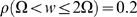 (i.e. ‘recent’ infections are less than 0.5 years old, and sensitivity to recent infections is equal to 1-specificity for infections between 0.5 and 1.0 years old [14]). [10], [14]. Beyond this time, PFP as a function of time since infection has not been directly observed, so we construct two hypothetical scenarios (Figure 1); it stays constant over time since infection at 5% (as implied by Hargrove *et al.* [14]: *Scenario A*), or it increases over time since infection to 50% after 20 years (*Scenario B*).

In the model analyses, the distribution of time since infection for the people living with HIV is described with respect to age and time for each of the six African countries considered. The extent of potential misclassification by the BED test was quantified as the proportion of individuals infected with HIV for at least one year that would be wrongly classified as recent infections, corresponding to the value of 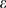 in Hargrove *et al.* The “Spectrum incidence rate” was compared with the estimate from the BED test, making alternative assumptions about the BED response function, and using the post-assay correction formulae assuming a constant value of 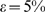. We note that the accuracy of the Spectrum incidence estimates compared to the real world is not known, but that, within this modelling exercise, they do represent the gold-standard against which to compare the corresponding simulation of the BED estimates. Indicative sample sizes required to statistically detect changes were calculated naively using standard formulae for differences in proportions [33].

## Results

**Figure 2:**
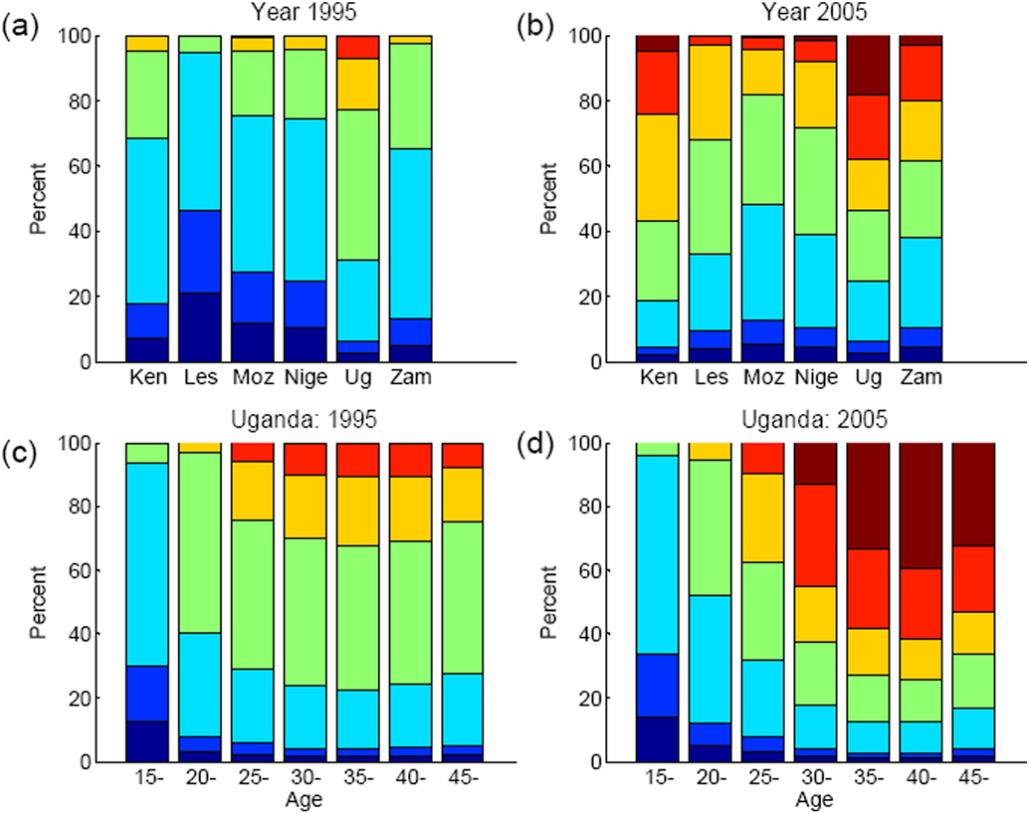
Distributions of time-since-infection for the HIV-infected populations across six African countries (Kenya, Lesotho, Mozambique, Nigeria, Uganda and Zambia) in (a) 1995 and (b) 2005; and across age-groups in Uganda in (c) 1995 and (d) 2005. Categories are (from base upwards): <4months (dark blue); <1 y (light blue); <4 y (cyan); <8 y (green); <12 y (yellow); <16 y(orange); 16+y (dark red).

Comparing the distribution of time since infection for those HIV infected by 1995 and ten years later, in 2005, there is a general increase in the proportion infected for a long time, but this varies greatly by country and age-group (Figure 2). Across countries, recent infections are more frequent in countries that have experienced recent epidemic growth (e.g. Mozambique) and late infections are more frequent following epidemic stabilisation and decline (e.g. Uganda and Kenya). Early in the epidemic, the distribution of time since infection is similar across all ages, but over time recent infections become relatively more common in young adults.

**Figure 3:**
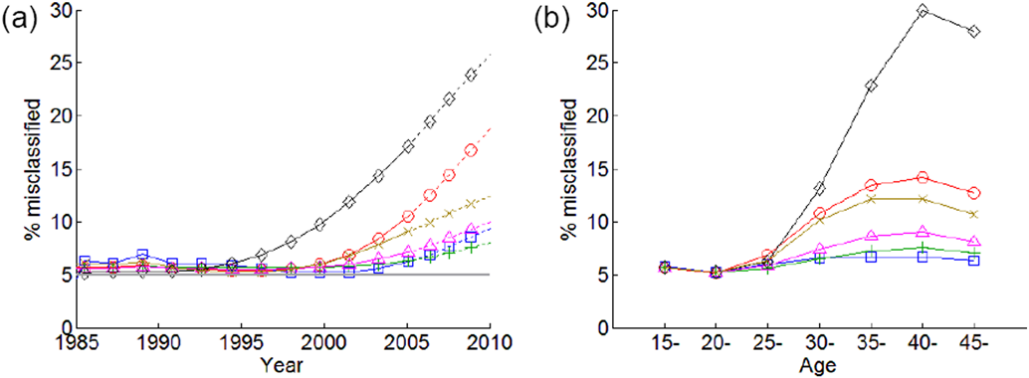
Proportions of infections at least one year old that are misclassified by the BED test for six African countries, (a) over time (ages 15–49), and (b) over age (in year 2005) using BED response scenario B (increasing proportion false positive: panels c & f). Lines show: Kenya (red line and circles), Lesotho (blue line and squares), Mozambique (green line and plus-signs), Nigeria (pink link and triangles), Uganda (black lines and diamonds), and Zambia (brown line and crosses). The lines are dotted after 2005, since Spectrum incidence were not calculated after 2005: these results are based on assuming incidence remains constant at 2005 levels.

These distributions results in substantial variation in the level of misclassification by the BED test, if the PFP increases with time since infection (scenario B in Figure 1). Figure 3 shows estimates of 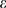, in the different countries over time (Figure 3(a)) and across age-groups in 2005 (Figure 3(b)). The extent of misclassification increases with time, especially in countries where the epidemic has declined. The rate of increase is related to the timing of epidemic spread, and varies between countries giving a range, in 2005, of 6% (Mozambique) to 17% (Uganda). Across age in 2005, the misclassification rate is stable (5%) for 15–24 year-olds, and sharply increases between ages 25–39 years (up to 30% in Uganda and 7% in Lesotho). However, this pattern with respect to age is dynamic, and the relative value of 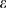 between the different age-groups also evolves over the course of the epidemic. The corresponding graphs for BED response scenario A shows a constant misclassification rate of 5%, as per the assumption.

The estimated incidence that would come from using the BED test, corrected using a constant value of 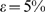 [14], is compared with the Spectrum incidence rate (Figure S2). For scenario A (PFP constant over time since infection) incidence is detected approximately correctly in all settings. In contrast, for Scenario B (PFP increases over time since infection), incidence is over-estimated in the period 1995–2005 in most countries. The extent of the over-estimate in 2005 is negligible in some countries (Lesotho and Mozambique – though it increases later) and highest in Kenya (3-times too high) and Uganda (5-times too high). Across countries, the extent of the bias with respect to age is highly variable. In general, the bias is modest in all settings among ages 15–29, but increase sharply at older ages, and is greatest for the 35–39 years age-group.

**Figure 4:**
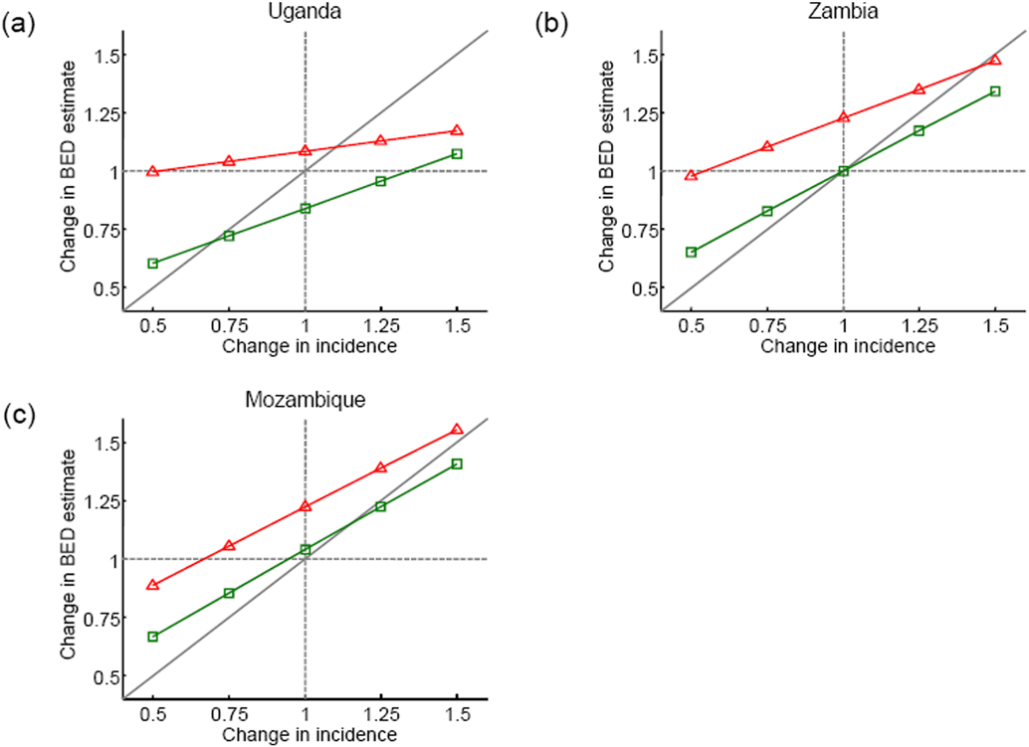
The ability of the BED test to detect changes in incidence. The incidence change estimated in 2006 five years after an instantaneous change in incidence by a factor 0.5, 0.75, 1.0, 1.25 or 1.5 (i.e. decreased by half, decreased by a quarter, no change, increased by a quarter, increased by half), using the alternative BED response scenarios: scenario A (stable proportion false positive: green squares); and, scenario B (increasing proportion false positive: red triangles). The analysis is repeated for incidence patterns representative of Uganda (a); Zambia (b); and, Mozambique (c). Note that a factor of 1.0 indicates no change in incidence. The thick diagonal grey line shows the 1∶1 relationship expected if the BED estimate perfectly tracks changes in incidence.

Figure 4 shows the relationship between trends in Spectrum and BED estimates of incidence following instantaneous changes in the incidence rate. For Uganda (a declining epidemic) and BED response scenario A, Spectrum trends in incidence are only roughly reflected in changes in the BED estimates (green line poorly approximates diagonal grey line) (Figure 4(a)). In the ‘no-change’ simulation (1.0 on the horizontal axis) the BED test falsely indicates a reduction in incidence, which is generated by the shifting distribution in time since infection among the infected population. For Scenario B, the relationship between actual changes in incidence and changes in the BED-estimates of incidence shows only a weak positive correlation. For Zambia and Mozambique (stable and growing epidemics, respectively), the performance of the BED estimates using scenario A is good, with no false detection of a trend in incidence and a close relationship between actual trends in incidence and that recorded in the BED test (Figure 4(b)). In Zambia and Mozambique there is a weak correlation between actual trends in incidence trends and changes in BED estimate using scenario B.

In stabilised epidemics, where our modelling indicates that actual changes in incidence could be reliably recorded in the BED estimates of incidence assuming scenario A, we calculated the sample sizes required to record a statistically significant difference in two cross-sectional sero-surveys. In these calculations, we require 80% chance of detecting a statistically significant difference in the proportion of recent infections in a two-sided test at the 5% significance level. For Zambia, where the rate of incidence is relatively high, sample sizes for each survey of approximately 12,000 and 54,000 are required to detect a 50% or 25% reduction in incidence respectively, assuming BED response function scenario A. For Nigeria, where the rate of incidence is much lower, sample sizes of approximately 52,000 and 220,000 would be required to detect a 50% or 25% reduction in incidence, assuming BED response function A. Sample sizes calculations using scenario B were not calculated since trends in BED estimates of incidence under these test properties could not be reliably interpreted.

## Discussion

Although it is widely recognised that the BED test can misclassify old infections as recent, there has been renewed confidence in using BED-derived estimate of incidence if a post-assay analytic correction procedure is used [14], [16]. However, for this to work, the proportion of non-recent infection that the test misclassifies (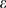) must be known. In this modelling exercise we have found that if the chance of misclassification by the test does not change over time, then empirical measurements of 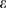 can be used universally, and corrected estimates of incidence will be accurate. However, we also find that if the chance of misclassification by the test increases for those with very advanced infections, then this quantity will vary by place, over time and across age-groups. It has been shown that using an incorrect value of 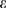 to adjust BED test results leads to substantial biases in ‘corrected’ estimates of incidence [14], [17]. Our modelling shows that comparisons of incidence estimates within populations (over age, or factors correlated with age such as marriage/widowhood), over time or between populations may also be unreliable.

The exact nature of the relationship between misclassification and time since infection is not known. There are observational studies showing that individuals with low CD4 counts/high viral loads are much more likely to be misclassified than infected individuals with high CD4 counts/low viral loads [19], [23], [24], [26]. Also, if the individuals with fully developed immune-responses that are misclassified as recent live for longer than others, then misclassification rates will also vary with time since infection [23], [28]. Moreover, the wide variation in the few empirical measurements of 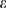 [13], [14], [17], [18], the greater values measured in Rwanda, Zambia, Uganda and Zimbabwe (where the epidemics have stabilised/declined) than South Africa (where the epidemic continues to grow) are consistent with the model predictions assuming that PFP does increase with time since infection. Furthermore, the empirical estimate of 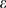 in Uganda [19] and the degree of apparent error in corrected BED estimate of incidence in Uganda and Kenya [21], [22], are also in close quantitative agreement with our model predictions. Thus, assuming that PFP is constant over time may not be safe, and reported estimates of incidence should properly reflect the uncertainty and potential for substantial error that would arise if PFP increases with time since infection.

Even though the level of the incidence estimate may be biased, it has been suggested that patterns with respect to age and time in BED estimates may used to identify risk groups and track changes in the epidemic. The BED test was recently used to identify groups at highest risk in Uganda [21], and the pattern of incidence over age was surprising: whilst these data suggested that the ages of peak incidence for women (35–39 years: 3.5/100pyar) was older than for men (30–34 years: 2.8/100pyar), other empirical data from Uganda and other countries in sub-Saharan African [34], [35], show that typically the age-groups with the highest incidence rates are ten years younger, and at older ages for men than women. The authors of a South African BED study also comment that incidence in older age-groups was surprisingly high [20]. In both cases, it was speculated that this may be due to patterns of widowing and remarriage, but our modelling indicates that this surprising pattern of incidence over age may, in fact, be due misclassification increasing with time infected.

We investigated the ability for the BED estimates of incidence to detect actual changes in incidence and found that, as anticipated by earlier observations [14], in some settings (e.g. Uganda and Kenya) declines in BED estimates of incidence can be spuriously generated by natural changes in the distribution of time-since-infection in the infected populations. In other settings (e.g. Zambia), we found that under certain assumptions about the BED response function (scenario A), changes in the BED estimate of incidence can reliably reflect changes in the actual incidence rate. However, the ability to record statistically significant changes in BED-estimates of incidence requires samples sizes much larger than are routinely used in existing surveys [21], [22], [36] (e.g. samples of ∼50,000 individuals per sero-survey in Zambia for a 25% reduction in incidence, and much more in settings with lower incidence). Another study has shown that when the uncertainty in any calibrating parameters is also considered, sample sizes of at least 10,000 are required even to identify greater changes in high-incidence settings (reviewed in [37]). Nonetheless, with a third survey a test for trend across time could be applied that may lead to smaller sample sizes being required in each survey. Official guidelines for the BED test do highlight the importance of using sufficiently large samples [16], but this poses substantial logistic difficulties in many settings.

Our modelling did not consider that the BED response scenario could also vary by sex, age, pregnancy status, or viral sub-types. All of these would lead to further variation in 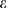 by place, time and age. It has also been shown that the measured sensitivity and specificity of the test over the first two years will depend on the distribution of time since infection [38], meaning that the biases in estimates could be even more varied than suggested here. We have not directly considered the use anti-retroviral treatment in the model, which can also lead to misclassification. While some national population-based surveys that have applied BED have not done this, future sero-surveys should identify those in treatment so that they could be excluded from the analyses. If the exclusion is not fully effective, treatment could introduce an additional time-variable bias, creating an exaggerated version of scenario B (Figure 1).

Our modelling supports empirical work showing the BED test can be used successfully with a locally and recently measured value of 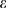 [14], [39]. However, we have also shown that the value of 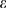 quickly becomes ‘out-of-date’, and cannot easily be transferred to other settings. Thus, for reliable results, 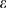 should be measured for each population in which the test is used. CD4 cell count data could help to exclude some individuals with long-term infections but, in addition to the logistical challenges of doing this, the variability in CD4 counts within and between individuals [40] would make such a correction technical involved and likely incomplete. Unfortunately, using reported behavioural data to exclude confirmed long-term infection (such as date of first positive HIV test) is not likely to lead to sufficient improvements in specificity, since testing rates in Africa are currently low [41].

The relationship between misclassification and time since infection could be directly observed by applying the BED assay (and other similar assays) to sera collected from individuals with known durations of infection ranging from 0 to 20 years. In addition, further immunological research may describe the underlying causes for changes in antibody composition over time, which could help inform the shape of the BED response function. This would provide the basis for making better assumptions made about the BED response function, enabling more reliable forms of *post hoc* correction.

From our analysis, we conclude that the current use and correction of BED test data could well lead to errors in understanding current patterns and trends in incidence. Much more work needs to be done to understand the test properties and such work should draw on longitudinal data from long-term cohort studies.

## Supporting Information

Text S1 (0.19 MB DOC)
