# Supplementary material for: Errors in ‘BED’-Derived Estimates of HIV Incidence Will Vary by Place, Time and Age
Source: PLoS One. 2009 May 28;4(5):e5720. doi: 10.1371/journal.pone.0005720 (PMC2684620; doi:10.1371/journal.pone.0005720)
Supplement: Text S1 — (0.19 MB DOC) [file pone.0005720.s001.doc]

**Appendix**

Numbers of susceptible ($X$) and infected ($Y$) individuals are tracked using a system of partial differential equations, with independent variables in calendar time ($t$), age ($a$) and time-since-infection ($w$):

$$\begin{matrix} \frac{\partial X_{k}(t,a)}{\partial t}+\frac{\partial X_{k}(t,a)}{\partial a}=-X_{k}(t,a)\left(\mu_{k}(a)+\lambda_{k}(t,a)\right) \\ \frac{\partial Y_{k}(t,a,w)}{\partial t}+\frac{\partial Y_{k}(t,a,w)}{\partial a}+\frac{\partial Y_{k}(t,a,w)}{\partial w}=-Y_{k}(t,a,w)\left(\mu_{k}(a)+h(a-w,w)\right) \\ \end{matrix}$$

where $k$is the sex (1: men; 2: women); $\mu_{k}(a)$is the background mortality rate; $\lambda_{k}(t,a)$is the incidence of HIV infection; and, $h(a_{0},w)$is the hazard of HIV-related causes of death for individuals infected at age $a_{0}$ that have survived for time$w$since infection. The following specify the boundary conditions for these equations (the unit of time is years):

Here, $S_{k}(a)$ is the number of individuals in each sex/age category at the start of the simulation, $q$is the fraction of individuals that survive from birth to exact age 15, and $\eta(a)$ is the per-woman fertility rate (Table S3).

The temporal trend in the force incidence rate ($\lambda_{k}(t,a)$) was estimated using Spectrum software and UNAIDS estimates of prevalence [1], which relate to the combined population of men and women aged 15-49 years ($f(t)$): see Figure S1. The pattern of incidence over age and sex was based on empirical estimates from rural Zimbabwe ($f_{k}(a)$): see Table S1. The age/sex pattern was assumed to be constant over time, so that: $\lambda_{k}(a,t)=f(t).f_{k}(a)$.

The calculation for the exact measurement of incidence at time $t$for ages in the range $A_{0}$ to $A_{1}$ is: $I_{e}(t)=\frac{\int_{A_{0}}^{A_{1}} \sum_{k} X_{k}(t,a)\lambda_{k}(t,a)da}{\int_{A_{0}}^{A_{1}} \sum_{k} X_{k}(t,a)da}$.

In this notation, the overall sensitivity ($\sigma(t)$) and specificity ($\pi(t)$) of the test vary by time and are given by:

$\sigma(t)=\frac{\int_{0}^{\Omega} \chi(w)N(t,w)dw\text{ }}{\int_{0}^{\Omega} N(t,w)dw}$ and $\pi(t)=\frac{\int_{\Omega}^{\infty} \left(1-\chi(w)\right)N(t,w)dw\text{ }}{\int_{\Omega}^{\infty} N(t,w)dw}$

where $N(t,w)=\int_{A_{0}}^{A_{1}} Y(t,a,w)da$.

The model is solved numerically using custom-written software in the *C++* programming language.

Following previously reported formulae for estimating incidence [2], the calculation for the ‘uncorrected’ BED measurement of incidence for ages in the range $A_{0}$ to $A_{1}$ at time $t$is:

$$\begin{matrix} I_{b}(t)=\frac{R(t)}{R(t)+\Omega\int_{A_{0}}^{A_{1}} \sum_{k} X_{k}(t,a)da} \\ R(t)=\int_{A_{0}}^{A_{1}} \int_{0}^{\infty} \chi(w)\sum_{k} Y_{k}(t,a,w).dw.da \end{matrix}$$

Where $\chi(w)$ is the BED response function (fraction of blood samples from individuals who have been infected for $w$years that the BED test classifies as recent), and $\Omega$ is the period for which new infections should be classified as recent by a perfect test.

The ‘corrected’ BED estimate (Hargrove et al.’s $\hat{I}_{II}$[3]) is:

$$I_{b}(t)=\frac{R(t)-\varepsilon\int_{A_{0}}^{A_{1}} \sum_{k} Y_{k}(t,a)da}{R(t)+\Omega\int_{A_{0}}^{A_{1}} \sum_{k} X_{k}(t,a)da-\varepsilon\left(\int_{A_{0}}^{A_{1}} \sum_{k} X_{k}(t,a)+Y_{k}(t,a)da\right)}$$

Where $\varepsilon=0.05$ is the proportion of individuals infected for more than a year misclassified as recent by the test, and $\Omega=0.5$.

**References**

**Figure S1**: Assumed HIV incidence trends (per 100 person-years at risk), 1985-2005, calculated using Spectrum and the UNAIDS estimates of HIV prevalence [4]. Note that although the estimated incidence trends may not exactly reflect actual rates in these populations, the intention here is to use a range of incidence patterns that are broadly representative of the epidemics observed in different parts of Africa. For the purposes of the modeling presented in this article, we refer to these incidence rates as the ‘Spectrum rates’, and are the gold-standard against which the BED estimates are compared.

**Figure S2:** Ratio of simulated *post-assay corrected* BED-estimates of incidence to ‘Spectruml’ incidence for six African countries over time (all ages; panels a-b) and over age (in year 2000: panels c-d) using alternative assumptions about the BED response relationship: scenario A (stable proportion false positive: panels **a & c**); and, scenario B (increasing proportion false positive: panels **b & d**). Lines show: Kenya (red line and circles), Lesotho (blue line and squares), Mozambique (green line and plus-signs), Nigeria (pink line and triangles), Uganda (black lines and diamonds), and Zambia (brown line and crosses). For the correction procedure, a constant value of 0.05 was used for the proportion of individuals infected for at least one year that are misclassified as recent by the test (which is correct for scenario B only).

| **Age-group** | **Relative Incidence Rate** | |
| --- | --- | --- |
|  | **Men** | **Women** |
| **15-19** | 0.43 | 1.19 |
| **20-24** | 0.72 | 1.19 |
| **25-29** | 1.33 | 1.23 |
| **30-34** | 1.16 | 1.06 |
| **35-39** | 1.16 | 0.78 |
| **40-44** | 1.08 | 0.60 |
| **45-49** | 0.99 | 0.42 |

**Table S1**: Pattern of incidence with respect to sex and age, based on recent empirical observations for rural eastern Zimbabwe. (Source: Lopman *et al.* [5]). This general pattern with respect to sex and age has also been observed in several other African countries [6]. Note that, in the model, this pattern is assumed to be constant over time.

| **Age-group** | **Shape (λ)** | **Scale (k)** | **Median Survival (years)** |
| --- | --- | --- | --- |
| **15-19** | 2.0 | 16.0 | 13.3 |
| **20-24** | 2.0 | 15.4 | 12.8 |
| **25-29** | 2.0 | 14.1 | 11.7 |
| **30-34** | 2.0 | 12.1 | 10.0 |
| **35-39** | 2.0 | 11.0 | 9.1 |
| **40-44** | 2.0 | 10.1 | 8.4 |
| **45-49** | 2.0 | 7.9 | 6.6 |

**Table S2**: Weibull distribution parameters describing net-survival with HIV by age at infection. (Source: Todd *et al.*, 2007 [7]). The Weibull survival distribution at time *t*, is computed as: *S(t)=exp(-(t/ λ)^k)*.

|  | **Population in 1980 (thousands)** | | **Background Mortality rate**  **(1980-2010)** | | **Fertility rate**  **(1980-2010)**  **(births per woman year)** |
| --- | --- | --- | --- | --- | --- |
| **Age-range** | **Men** | **Women** | **Men** | **Women** |  |
| **15-19** | 373 | 365 | 0.004 | 0.004 | 0.175 |
| **20-24** | 304 | 299 | 0.006 | 0.005 | 0.313 |
| **25-29** | 247 | 244 | 0.007 | 0.006 | 0.324 |
| **30-34** | 200 | 198 | 0.007 | 0.006 | 0.271 |
| **35-39** | 161 | 161 | 0.008 | 0.007 | 0.201 |
| **40-44** | 129 | 129 | 0.010 | 0.008 | 0.125 |
| **45-49** | 102 | 103 | 0.012 | 0.009 | 0.053 |

**Table S3**: Demographic assumptions used in the model (Source: World Bank: World development report-1991 [8]). On the basis of the same data source, it was also assumed that survival from birth to exact age 15, $q$, was 0.78. The model is run for 50 years before starting the simulation so that a stable demographic structure is established. In the absence of HIV, the population grows at a constant rate of 3.5%. Note that the same demographic assumptions are used in model simulations for all countries. Sensitivity analysis indicates that using an alternative set of demographic assumptions does not materially affect the results presented (not shown: available from authors on request).
